# Long-term patient-reported outcomes in pediatric partial-thickness burns using Epiprotect® and Biobrane® a retrospective comparative study

**DOI:** 10.1016/j.jpra.2025.11.002

**Published:** 2025-11-08

**Authors:** Olivia J. Hartrick, Bryant Chong, Ferdinand B. Mayer, Suraya M. Yusuf, Rhiannon E. Cope, Ankit Mishra, Ameer Khamise, Alexandra Murray

**Affiliations:** a Plastic Surgery Department, Stoke Mandeville Hospital, Mandeville Road, Aylesbury, Buckinghamshire HP21 8AL, United Kingdom; b NDORMS, Botnar Institute for Musculoskeletal Sciences, University of Oxford, Windmill Road, Oxford, Oxfordshire OX3 7LD, United Kingdom; c The University of Buckingham, Department of Medicine, Hunter Street, Buckingham, Buckinghamshire MK18 1EG, United Kingdom

**Keywords:** Burn, Pediatric, Epiprotect®, Biobrane®, PROMS, Long-term outcomes

## Abstract

**Background:**

Larger pediatric partial-thickness (PT) burns are typically managed with debridement and a skin substitute. Epiprotect® has shown comparable acute surgical outcomes and lower infection rates than Biobrane®, but data on long-term outcomes remain limited.

**Purpose:**

To compare long-term patient-reported outcomes between Biobrane® and Epiprotect® in pediatric PT burns using the Brisbane Burn Scar Impact Profile (BBSIP).

**Methods:**

We included all pediatric patients (<18 years) treated with Biobrane® or Epiprotect® for PT burns at our center between February 2018 and July 2023 who completed the BBSIP survey >12 months after injury. Total BBSIP scores were analyzed using Wilcoxon rank-sum tests and a negative binomial regression, adjusting for total body surface area (TBSA) and burn depth.

**Results:**

Forty-eight patients were included (Biobrane® *n* = 32; Epiprotect® *n* = 16). Median age was 21 months (8 months–13 years); mean TBSA was 6 % (2–15 %). Total BBSIP scores were significantly better in the Epiprotect® group (median = 7.74) compared to Biobrane® (median = 8.06; *P* = 0.0145). In the multivariable analysis, Epiprotect® was associated with lower total BBSIP scores compared with Biobrane® (β = –0.22, *P* = 0.04) adjusting for TBSA (β = 0.059, *P* < 0.001) and burn depth (β = –0.26, *P* = 0.006).

**Conclusions:**

Epiprotect® was associated with improved long-term outcomes compared to Biobrane®, particularly in larger or deeper burns. These findings support its use when long-term function and well-being are prioritized.

## Introduction

Larger pediatric partial thickness (PT) burns are commonly managed with debridement and the application of a skin substitute. Epiprotect® and Biobrane® are biosynthetic skin substitutes used in burn care; Biobrane® consists of a silicone membrane bonded to a nylon mesh coated with porcine collagen, while Epiprotect® is a cellulose-based skin substitute. Previous research has indicated that Epiprotect® has comparable acute surgical and healing outcomes but lower infection rate compared to Biobrane®.^1^, ^2^, ^3^, ^4^ However, reports on long-term outcomes of these two skin substitutes are limited.^5^ Clinical outcome reporting in burn care is substantially heterogenous.^6^ This inconsistency in reporting impedes the effective synthesis of data, which is essential for clinical decision-making. An international consensus led to the development of a core outcome set for burn surgery to standardize reporting of long-term outcomes. More recently, the identification of the top ten global research priorities in burns care has further emphasized the importance of high-quality evidence on patient-reported outcomes to answer these priorities.^7^^,^^8^ The Brisbane Burn Scar Impact Profile (BBSIP) is a validated patient-reported outcome measure (PROM) which can be used to collect some of these core outcomes: the ability to do daily tasks; neuropathic pain and itch and psychological wellbeing.^9^ This retrospective study provides a multivariable regression analysis of the long-term patient-reported outcomes following the application of Biobrane® and Epiprotect® in pediatric patients burns.

### Purpose of the study

To compare the long-term patient-reported outcomes of pediatric patients who are treated with Biobrane® and Epiprotect®. We hypothesize that these two skin substitutes have comparable results.

## Methods

This retrospective observational study compared the clinical outcomes of pediatric patients treated at the Pediatric Burns Unit of Stoke Mandeville Hospital from May 2018 to July 2023 who sustained PT burns and were managed with Biobrane® or Epiprotect® . Data acquisition was conducted initially by the local data support analyst (RW) and then systematically by authors who retrieved electronic medical records from the hospital. All pediatric patients (age <18 years) with various degrees of PT burns, including superficial partial thickness (SPT), deep partial thickness (DPT) or a combination of both who were managed with Biobrane® or Epiprotect® who had completed the BBSIP survey for caregivers >12 months after their initial injury were included. Patients aged 18 or over or with insufficient data in the hospital’s medical records were excluded (Table 1).

**Table 1:** Inclusion and exclusion criteria.

Inclusion criteria	Exclusion criteria
• All pediatric patients below the age of 18 years old	• 18 years or older
• Superficial partial thickness (SPT) burns, Deep partial thickness (DPT) burns, or a combination of both	• Burns managed with both Epiprotect® and Biobrane®
• Total or partial coverage with either Epiprotect® or Biobrane®	• Incomplete BBSIP patient-reported outcome measure data

Burn depth was assessed based on clinical judgement by the admitting plastic surgeon. Dressing selection for wound treatment was determined by the surgical team pre- and post-burn debridement. In 2020, the treatment approach transitioned from Biobrane® to Epiprotect®, primarily due to supply-chain issues.^4^

### Clinical method

**Figure 1:**
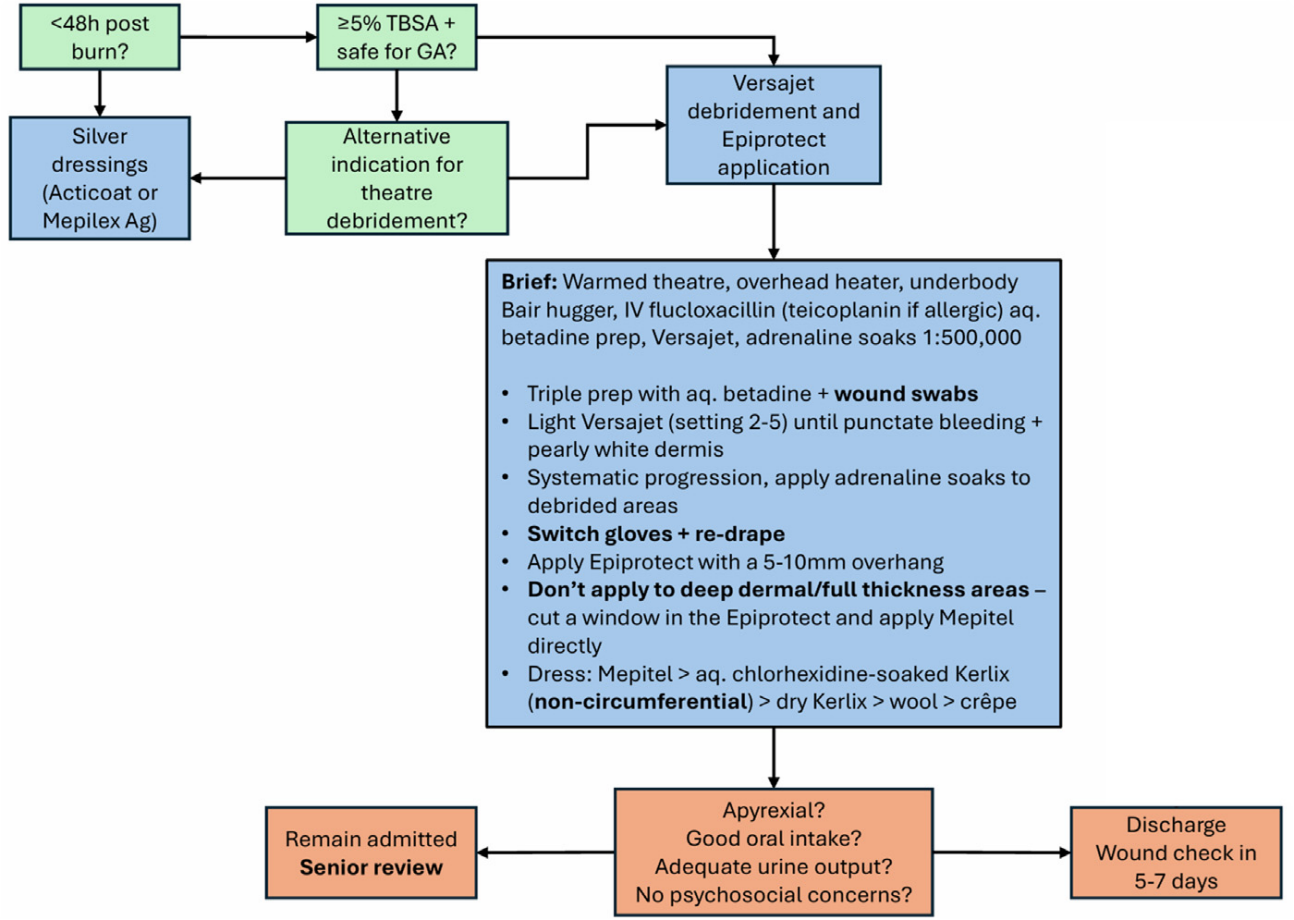
The inpatient Epiprotect® protocol for Pediatric burns at the Pediatric Burns Unit of Stoke Mandeville Hospital.

As previously documented in our preceding paper which reported the acute healing outcomes,^4^ there was no difference in clinical management between the Biobrane® and Epiprotect® groups. Adequate first aid was defined as the application of cool or lukewarm running water for at least 20 min within 3 h of injury, and with removal of clothing and jewelry. In this pediatric burns unit, patients with ≥5 % TBSA confluent area of superficial partial thickness (SPT) burn are considered for early debridement in theatre. This ideally will occur within 24 h but may occur within the first few days after injury. The assessment of burn depth is carried out clinically. Burn size (total body surface area, TBSA) was assessed clinically using the pediatric Lund & Browder chart, which is standard practice in our unit. Debridement under general anesthesia may still be considered for children with <5 % TBSA burns in cases where there are high levels of distress, complex or deeper injury patterns or signs of infection. However, for most <5 % TBSA burns, the additional benefit of exudate control, improved pain control and healing gained from using skin substitutes is not felt to warrant the risks of general anesthesia. The inpatient protocol for Epiprotect® is outlined in Figure 1. Cases of mixed-depth burns may be treated using a mixture of skin substitutes with conventional dressings. While our use of skin substitutes is primarily reserved for SPT burns, they are also used for deep partial thickness (DPT) wounds (i.e., mid-dermal or deep dermal) where it is felt that meticulous dressings in a well-optimized patient with residual viable dermis may avoid the need for skin grafting. All cases in this study underwent skin substitute application under general anesthesia in an operating theatre. All patients were given antibiotics upon induction (flucloxacillin or teicoplanin in case of penicillin allergy). The burns are prepared with warmed aqueous betadine solution and are then debrided using a Versajet device (Versajet II® Hydrosurgery system, Smith & Nephew, Watford, UK). For SPT burns, debridement is continued until the dermis takes on a healthy, pearlescent white appearance with light bleeding. For DPT injuries, the endpoint of excision is punctate bleeding. Swabs soaked in 1:500,000 adrenaline solution are used to maintain hemostasis. Once the wound is fully debrided, new drapes and gloves are used, and the skin substitute is laid on the burn. It is trimmed to 5 mm beyond the border of the burn. Then, Biobrane®, or Epiprotect® more recently are affixed with either tissue adhesive or suture strips where needed. A dressing consisting of Kerlix™ soaked in aqueous chlorhexidine gluconate solution, dry Kerlix™, wool and crepe is then applied. The use of routine post-operative antibiotics is left to the discretion of the operating surgeon and is usually given only if the wound is evidently sloughy, the child has been pyrexial or surgical intervention occurs beyond 48 h. Patients remain for at least one night in the hospital to ensure the child remains apyrexial, comfortable, is eating and drinking well and dressings have remained intact. This also allows the patient and family to be seen by the multi-disciplinary team, including psychologist, physiotherapist and dietician, prior to discharge. The first wound inspection takes place 5–7 days post-surgery and is usually carried out in a pediatric outpatient setting. For larger injuries or in very distressed children, this may be performed under sedation in the ward, or occasionally in the theatre under general anesthetic. Thereafter, wound inspections are performed every 5–7 days in the burn’s clinic until healed and then patients are referred to scar clinic for ongoing follow-up according to clinical need and after 12 months, patients and caregivers completed the BBSIP survey, which was administered via Microsoft Forms.

In rare cases of <5 % TBSA burns with complex patterns or high levels of distress, substitutes were used following clinical judgement; such cases were included if they met study criteria. No patients subsequently required grafting or Recell within this cohort, and all progressed to healing with conservative management following substitute application.

### Statistical method

We extracted data on a range of clinical and demographic variables, including date of injury, age at the time of injury, sex, mechanism of injury, total body surface area (TBSA) affected, burn depth (classified as superficial partial thickness [SPT], deep partial thickness [DPT], or mixed), the type of skin substitute used (Biobrane® or Epiprotect®), the day of application in relation to the date of injury, and the total Brisbane Burn Scar Impact Profile (BBSIP) score recorded >12 months after injury. All burns in this cohort were scald injuries.

The primary outcome measure was the total score from the Brisbane Burn Scar Impact Profile (BBSIP), a validated caregiver-reported instrument for pediatric burn survivors. The BBSIP includes eight domains: sensory symptoms, daily activities, appearance, mobility, emotional reactions, social participation, physical symptoms, and parent concerns. Scores within each domain range from 1 to 5, and a total score is obtained by summing all domain scores. Higher scores indicate greater scar impact and therefore worse patient-reported outcome.

Descriptive statistics were used to summarize all variables. As the data were non-parametric, initial comparisons between treatment groups were made using the Wilcoxon rank-sum test (equivalent to the Mann–Whitney U test).

To explore the relationship between treatment type and long-term outcomes, we performed a negative binomial generalized linear model (GLM) using total BBSIP score as the dependent variable. An initial full model included all relevant covariates: age at injury, TBSA, burn depth, and timing of skin substitute application. We then used backwards stepwise elimination to remove non-significant variables one by one, resulting in a final model that retained only those with a significant (*P* < 0.05) predictors. Model diagnostics were carried out to assess fit and check for overdispersion.

All statistical analyses were carried out using R (version 4.5.0). This study follows the Strengthening the Reporting of Observational Studies in Epidemiology (STROBE) guidelines for reporting observational research.

## Results

Out of 99 patients screened, 48 were included in the final analysis. Patients were excluded due to incomplete outcome data (*n* = 42) or missing key clinical variables (*n* = 9). All included patients had complete data available for skin substitute type, age at injury, burn depth, TBSA, day of application, and total score. Thirty two patients who received Biobrane® and 16 patients who received Epiprotect® were included. The median age at injury was 20 months (8–124) in the Biobrane® group and 22 months (9–157) in the Epiprotect® group. The distribution of sex was 15 males and 17 females in the Biobrane® group, and 10 males and 6 females in the Epiprotect® group (Table 2).

**Table 2:** Comparison of patient characteristics between Biobrane® and Epiprotect® groups.

Characteristics	Biobrane®	Epiprotect®
Total number included	32	16
Sex (male/female)	15/17	10/6
Age (months)	20 (8–124)	22 (9–157)
TBSA (%)	6.0 (2–15)	6.0 (2–12)
Number of mixed-depth burns (%)	17 (53.1 %)	10 (62.5 %)
Adequate first aid (%)	12 (37.5 %)	6 (37.5 %)
Day of application	1 (0–2)	1.5 (1–3)
Post operative antibiotics (%)	17 (53.1 %)	6 (37.5 %)

Data shown as n (%) or median (minimum − maximum); TBSA, total body surface area; If first aid was “unknown,” it has been classified as “inadequate.” Values for age show the median (minimum − maximum).

The mean TBSA burned was 6.6 % (2–15 %) in the Biobrane® group and 6.0 % (2–12 %) in the Epiprotect® group. Mixed-depth burns were present in 17 cases in the Biobrane® and 10 in Epiprotect® group (Table 3).

**Table 3:** Comparison of burn depth between Biobrane® and Epiprotect® groups.

Depth	Biobrane®	Epiprotect®
SPT	15 (46.9 %)	6 (37.5 %)
DPT	0 (0.0 %)	0 (0.0 %)
MD	17 (53.1 %)	10 (62.5 %)

Data shown as n (%); SPT, superficial partial thickness; DPT, deep partial thickness; MD, mixed depth (wound with both SPT and DPT burns).

No cases of substitute loss due to infection were identified in either group, and no major post-operative complications were recorded. Of note, 4 participants overlapped with the “infected” group described previously.^4^

The primary outcome was the total BBSIP score, a composite of eight caregiver-rated domain scores. The total BBSIP score was significantly different between treatment groups. The median total score was 8.06 in the Biobrane® group and 7.74 in the Epiprotect® group. A Wilcoxon rank-sum test indicated this difference was statistically significant (*W* = 367.5, *P* = 0.0145). This finding implies a potential difference in overall long-term outcomes between Epiprotect® and Biobrane®, although the clinical significance of this difference should be interpreted alongside adjusted analyses and the score context. Domain-level BBSIP scores for each treatment group are provided in Supplementary Table S1. No individual domains differed significantly between groups, consistent with the total BBSIP results.

**Figure 2:**
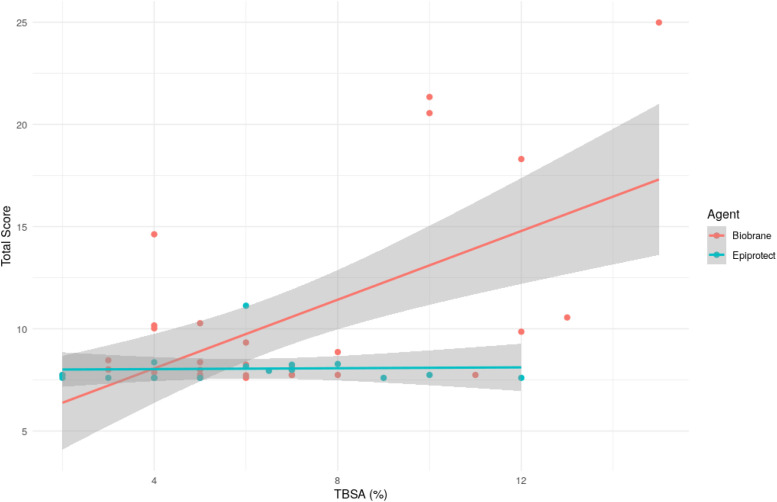
This plot shows the mean total BBSIP scores per agent and the percentage total body surface area, with confidence intervals 95 %.

**Figure 3:**
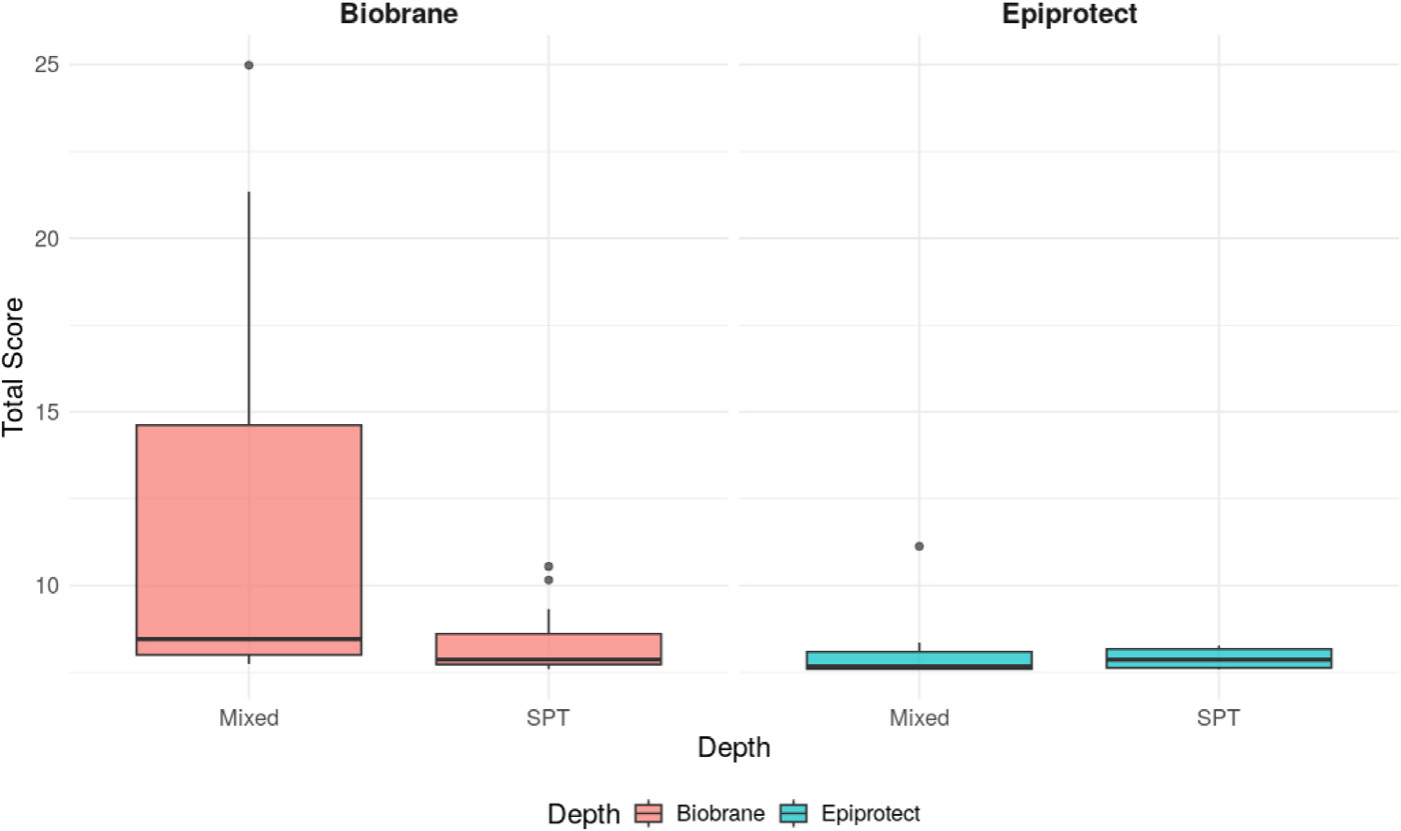
Box plots showing the distribution of mean total BBSIP scores by burn depth and treatment type. Boxes represent the interquartile range (IQR), horizontal lines indicate the median, and whiskers extend to 1.5× the IQR. Mean values are shown as points within each box.

A negative binomial regression model was fitted to examine the association between skin substitute type and total score, adjusting for TBSA and burn depth. In this model, TBSA remained a strong independent predictor of outcome: each 1 % increase in TBSA was associated with a 6 % increase in the expected total score (β = 0.059, *P* < 0.001). In addition, superficial partial-thickness burns were associated with significantly lower scores than mixed-depth burns (β = –0.26, *P* = 0.006). Patients treated with Epiprotect® reported lower scores compared to patients treated with Biobrane® overall (β = –0.22, *P* = 0.04) (Figures 2 and 3).

These findings suggest that skin substitute type, burn size, and burn depth all independently influence total score, with Epiprotect® associated with modestly lower scores compared to Biobrane® after accounting for TBSA and burn depth. However, there was no significant difference between the treatment groups after accounting for age at the time of injury and when the skin substitute was applied. Post-operative antibiotic use did not differ significantly between treatment groups and showed no significant association with total BBSIP scores in univariable or multivariable analyses.

## Discussion

### Key results

This retrospective cohort study compared long-term outcomes between pediatric patients with partial-thickness burns treated with either Biobrane® or Epiprotect®. Contrary to our hypothesis, we found that Epiprotect® was associated with statistically significant lower BBSIP scores and therefore better outcomes than Biobrane®, even after adjusting for TBSA and burn depth. Larger TBSA and deeper burns were also independently associated with higher scores, suggesting worse outcomes in those groups.

### Limitations

This study has several limitations that should be considered when interpreting the findings. First, the retrospective design introduces potential for selection and information bias. Treatment allocation was not randomized but determined by clinical teams, and Biobrane® was used earlier in the study period than Epiprotect®, raising the possibility of temporal confounding due to changes in practice, team experience, or follow-up quality. However, all participants completed the BBSIP at least 12 months post-injury, and multidisciplinary care pathways and scar management protocols remained consistent throughout the study period, which helps to mitigate this potential bias. Second, while BBSIP is a validated outcome tool, it was only administered once, >12 months after injury, and may be influenced by recall bias or unrelated life events. Additionally, the relatively small sample size, particularly for the Epiprotect® group (*n* = 16), limits statistical power and may increase the risk of Type I or Type II errors. Lastly, although the regression model adjusted for important clinical variables such as TBSA and burn depth, other potential confounders, such as dressing change frequency, or psychosocial factors, were not available. In addition, Fitzpatrick skin type was not consistently recorded in the retrospective dataset and could not be analyzed, and objective burn depth assessments (e.g., laser Doppler imaging) were not routinely employed in our pediatric pathway. These factors may have influenced outcomes and should be considered in future prospective studies.

### Interpretation

These findings suggest that Epiprotect® may provide better long-term outcomes than Biobrane® in pediatric partial-thickness burns. This complements previous studies that reported favorable short-term outcomes with Epiprotect®, including lower infection rates and similar healing times.^4^ Our results extend this evidence base by showing a potential advantage in terms of long-term function, comfort, and psychosocial well-being. Importantly, burn size and depth remained strong predictors of outcome, reinforcing the need to stratify treatment evaluations by injury severity. While the magnitude of benefit observed with Epiprotect® was modest, it may be clinically meaningful given the importance of functional recovery and quality of life in pediatric patients. This study also supports the internationally agreed core outcome set for burn care and aligns with recent global research priorities, which highlight the importance of assessing long-term patient-reported outcomes, particularly in children.^7^^,^^8^

### Generalizability

These results may be cautiously generalized to other UK pediatric burn units with similar surgical protocols and follow-up practices. However, the single-center design, modest sample size, and retrospective nature of the study limit broader applicability. Multicenter, prospective studies using standardized outcome sets such as the BBSIP would help to validate these findings and clarify the longer-term impact of skin substitute selection on recovery.

## Conclusions

Our findings demonstrate that long-term patient-reported outcomes with Epiprotect® were significantly better than those with Biobrane® in pediatric partial-thickness burns. This difference was particularly notable in patients with larger or deeper burns, where Biobrane® was associated with worse BBSIP scores. These results reinforce the value of standardized long-term outcome reporting and contribute to addressing recognized global research priorities in pediatric burn care.

## Declaration of AI technologies in the writing process

During the preparation of this work the authors occasionally used ChatGPT 4o to improve the readability and language of some sentences. After using this tool, the authors reviewed and edited the content as needed and take full responsibility for the content of the publication.

## Funding

None.

## Ethical approval

Not required.

## Declaration of competing interest

None declared.

## Appendix Supplementary materials

Image, application 1
