# Supplementary material for: Long-term patient-reported outcomes in pediatric partial-thickness burns using Epiprotect® and Biobrane® a retrospective comparative study
Source: JPRAS Open. 2025 Nov 8;48:56–64. doi: 10.1016/j.jpra.2025.11.002 (PMC12686885; doi:10.1016/j.jpra.2025.11.002)
Supplement: Supplementary file 1 [file mmc1.docx]

**Long-term patient-reported outcomes in paediatric partial-thickness burns using Epiprotect® and Biobrane®** ​**a retrospective comparative study**.

Miss Olivia J Hartrick^a, b*^, Dr Bryant Chong^a^, Dr Ferdinand B Mayer^a^, Miss Suraya M Yusuf^a^, Dr Rhiannon E Cope^a^, Mr Ankit Mishra^a^ Mr Ameer Khamise^c^, Miss Alexandra Murray^a^

Supplementary table

| **Table S1:** Domain-level BBSIP results by treatment group and between-group comparison | | | | |
| --- | --- | --- | --- | --- |
| Domain | Epiprotect | Biobrane | Coefficient | *P-*value |
|  | *Mean (± SD)* | *Mean (± SD)* | *Mean (± SE)* |  |
| 1 | 1.05 (0.09) | 1.41 (0.82) | -0.3 (± 0.30) | 0.293 |
| 2 | 0.71 (0.40) | 1.04 (0.94) | -0.38 (±0.34) | 0.266 |
| 3 | 1 (0.00) | 1.12 (0.32) | -0.11 (±0.30) | 0.714 |
| 4 | 1.02 (0.08) | 1.18 (0.60) | -0.14 (±0.30) | 0.631 |
| 5 | 1.05 (0.14) | 1.53 (1.00) | -0.38 (±0.28) | 0.179 |
| 6 | 1.01 (0.04) | 1.18 (0.48) | -0.16 (±0.30) | 0.589 |
| 7 | 1.14 (0.17) | 1.45 (0.59) | -0.24 (±0.28) | 0.394 |
| 8 | 1.07 (0.22) | 1.30 (0.54) | -0.2 (±0.29) | 0.494 |
| BBSIP = Brisbane Burn Scar Impact Profile. Data are presented as mean ± standard deviation (SD) for each domain, with coefficients (± standard error, SE) and p-values representing between-group comparisons (Epiprotect® vs. Biobrane®). No domain-level differences reached statistical significance. | | | | |
